# Supplementary figures and images for: Asymptomatic Malaria Reservoirs in Honduras: A Challenge for Elimination
Source: Pathogens. 2024 Jun 27;13(7):541. doi: 10.3390/pathogens13070541 (PMC11280452; doi:10.3390/pathogens13070541)

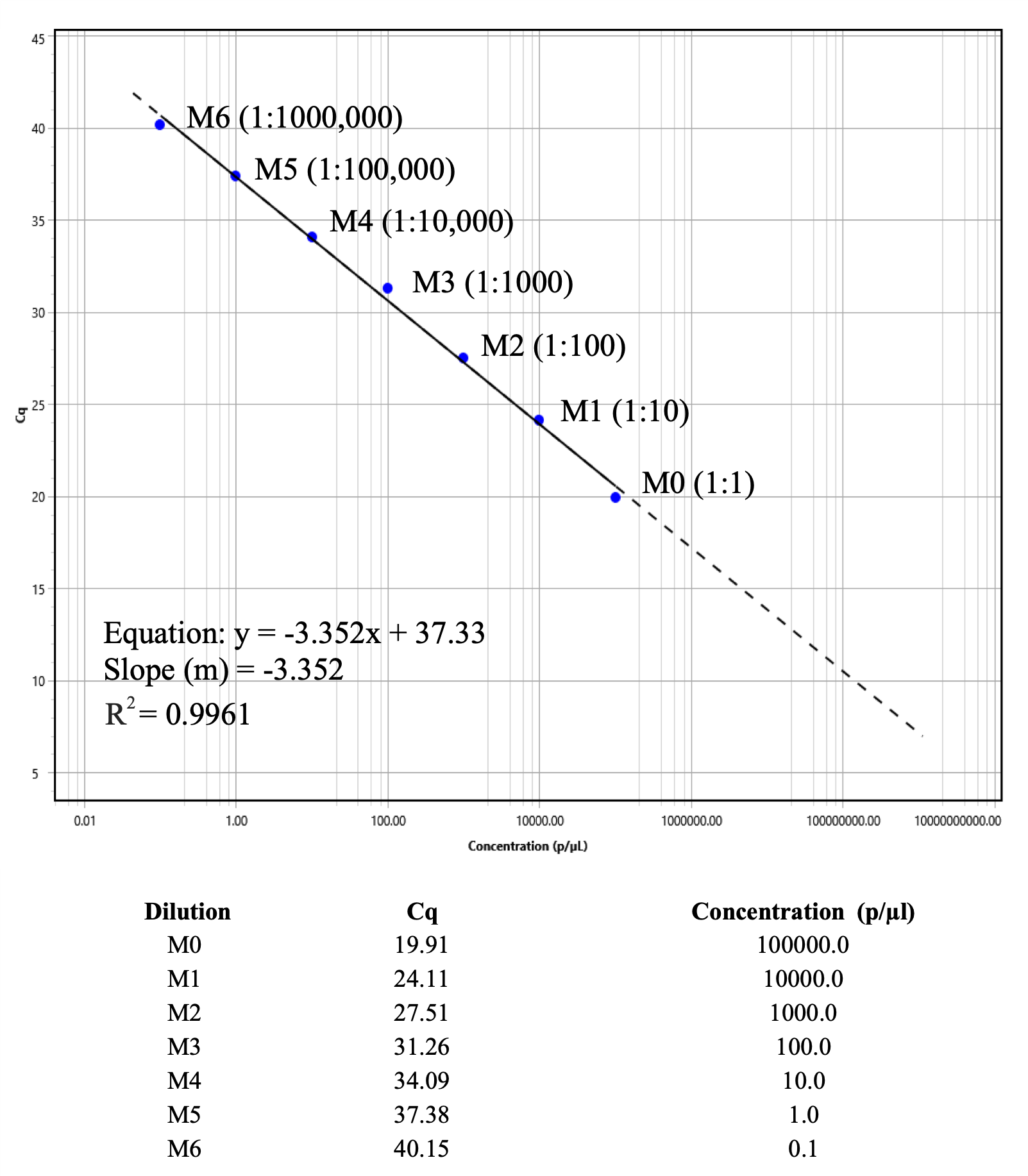

Supplement: Supplementary file 1 [file pathogens-13-00541-s001.zip › Supplementary Figure S1.png]

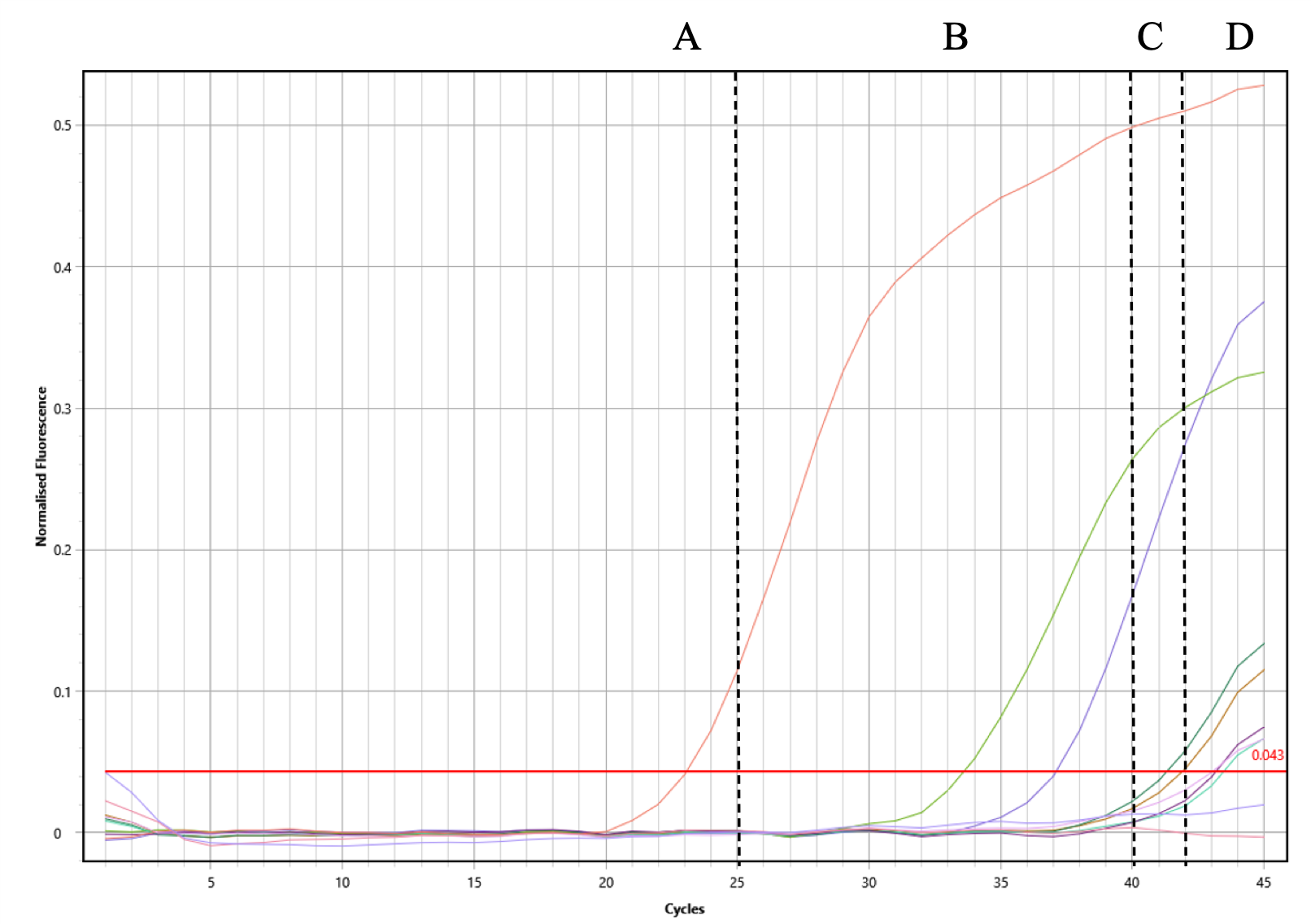

Supplement: Supplementary file 1 [file pathogens-13-00541-s001.zip › Supplementary Figure S2.png]
